# Highly N_2_-Selective Activated Carbon-Supported Pt-In Catalysts for the Reduction of Nitrites in Water

**DOI:** 10.3389/fchem.2021.733881

**Published:** 2021-08-05

**Authors:** Olívia Salomé G. P. Soares, Erika O. Jardim, Enrique V. Ramos-Fernandez, Juan J. Villora-Picó, M. Mercedes Pastor-Blas, Joaquín Silvestre-Albero, José J. M. Órfão, Manuel Fernando R. Pereira, Antonio Sepúlveda-Escribano

**Affiliations:** ^1^ Laboratório de Catálise e Materiais (LCM), Laboratório Associado LSRE-LCM, Departamento de Engenharia Química, Faculdade de Engenharia, Universidade do Porto, Porto, Portugal; ^2^ Laboratorio de Materiales Avanzados, Departamento de Química Inorgánica, Instituto Universitario de Materiales de Alicante, Universidad de Alicante, Alicante, Spain

**Keywords:** nitrite reduction, Pt-In catalysts, activated carbon, XPS, temperature-programmed reduction

## Abstract

The catalytic reduction of nitrites over Pt-In catalysts supported on activated carbon has been studied in a semi-batch reactor, at room temperature and atmospheric pressure, and using hydrogen as the reducing agent. The influence of the indium content on the activity and selectivity was evaluated. Monometallic Pt catalysts are very active for nitrite reduction, but the addition of up to 1 wt% of indium significantly increases the nitrogen selectivity from 0 to 96%. The decrease in the accessible noble metal surface area reduces the amount of hydrogen available at the catalyst surface, this favoring the combination of nitrogen-containing intermediate molecules to promote the formation of N_2_ instead of being deeply hydrogenated into NH^4+^. Several activated carbon-supported Pt-In catalysts, activated under different calcination and reduction temperatures, have been also evaluated in nitrite reduction. The catalyst calcined and reduced at 400°C showed the best performance considering both the activity and the selectivity to nitrogen. This enhanced selectivity is ascribed to the formation of Pt-In alloy. The electronic properties of Pt change upon alloy formation, as it is demonstrated by XPS.

## Introduction

Although nitrate occurs naturally in some groundwater, its presence in drinking-water is frequently associated with contamination by excessive use of fertilizers, in combination with inappropriate farming practices and/or sewage disposal (World Health Organization, 2011). The use of nitrates in agriculture as organic or chemical fertilizers has been a major source of water pollution in Europe. Nitrate is not directly toxic to humans, but under oxygen-free conditions such as in the human gut it is converted to nitrite. Nitrite can pass from the gut into the blood stream where it bonds to haemoglobin molecules, converting them to a form that cannot transport oxygen, methaemoglobin, causing the so called “blue baby syndrome” (methahemoglobinemia) (Comly, 1945). In addition, nitrate is a precursor of carcinogenic nitrosamines (Taylor, 1983). In an attempt to avoid the risks derived from the presence of nitrates in drinking water, the World Health Organization (WHO) has established a maximum contaminant level of 50 mg L^−1^, whereas the U.S. Environmental Protection Agency (USEPA) (World Health Organization, 2011) has lowered it up to 10 mg L^−1^

The removal of nitrate from drinking water is necessary in order to protect the environment and human health. The technologies for their removal are usually more complex and more expensive than those required for microbial control. Nevertheless, the increasing pollution of natural sources of drinking water encourages the development of new emerging technologies and processes for water remediation. The catalytic reduction by using hydrogen over a solid catalyst is a promising technique for the nitrate/nitrite removal. The main drawback of this process is the formation of ammonia as by-product, which is undesirable in drinking water (Anoop and Viraraghavan, 1997; Pintar, 2003).

Nitrate reduction can be described by a series of consecutive and parallel reactions where nitrate is reduced to nitrite in the presence of hydrogen, which is then converted to nitrogen as main product and ammonia as undesired by-product (Hörold et al., 1993a; Prüsse and Vorlop, 2001; Yoshinaga et al., 2002). The nitrite reduction seems to play an important role in the process selectivity (Yoshinaga et al., 2002). Only a few research works have been published on the reduction of nitrite in water, and most of the work done in this area is related to nitrate reduction. However, as nitrite is the intermediate product in nitrate reduction, it is important to evaluate catalysts that allow for understanding the process of its selective reduction to nitrogen. It has been reported (Hörold et al., 1993b; Soares et al., 2008) that several noble metals (Pd, Pt, Ru, Ir, Rh) are effective catalysts for nitrite reduction; nevertheless, among them only Pd-based catalysts show high activity and nitrogen selectivity. It has been also reported that nitrite reduction is influenced by reaction conditions (Hörold et al., 1993b), the metal catalyst particle size (Strukul et al., 1996) and also by the support used (Chinthaginjala and Lefferts, 2010). Most of the works published related to nitrite reduction have been carried out over monometallic catalyst (Höller et al., 2001; Chinthaginjala and Lefferts, 2010), only some of them being based on bimetallic systems (Witońska et al., 2008).

It has been shown that the selectivity to ammonia during nitrate reduction is higher when the noble metal is isolated, because as the noble metal is very active for hydrogenation reactions, the nitrite molecules formed are deeply hydrogenated into ammonia. In the present study, the effect of the indium content on the performance of activated carbon-supported Pt-In catalysts has been investigated in the nitrite reduction reaction, taking into consideration both the catalytic activity and the selectivity to nitrogen. It is expected that the addition of indium to Pt can increase the selectivity of the process towards nitrogen. There are some information in the literature concerning the utilization of supported Pd-In catalysts in nitrate reduction (Witońska et al., 2008; Soares et al., 2009; Marchesini et al., 2010). In a recent study, Durkin et al. reported the utilization of a lignocellulose-supported Pd-In catalyst for nitrate reduction (Durkin et al., 2018), and claimed that it was 10 times more active and air-stable than Pd-Cu catalysts prepared in a similar way. However, there are only a few studies on the utilization of Pt-In catalysts in this process, and most of them use metal oxides as catalytic supports (Marchesini et al., 2008). In this study, several activated carbon-supported Pt-In catalysts with different metal atomic ratios and activated under different calcination and reduction temperatures, have been tested in the reduction of nitrites in water with hydrogen. To the best of our knowledge, this is the first systematic study using activated carbon as catalyst support for this Pt-In system.

## Experimental

### Catalysts Preparation

A commercial activated carbon supplied by Mead Westvaco (Nuchar RGC30), with 0.5–1.0 mm particle size, was used as initial support. The carbon was first treated at 100°C in inert atmosphere overnight, in order to remove the adsorbed water. Pt and In were co-impregnated using an aqueous solution (10 ml of solution per gram of activated carbon) of H_2_PtCl_6_·6H_2_O and In(NO_3_)_3_·5H_2_O, respectively, with the proper amount of precursors to achieve the desired loadings. Once the carbon was added to the solution, it was kept under stirring for 12 h and, finally, the solvent was evaporated at 100°C in a rotary evaporator until dryness. Then, the samples were heat-treated in N_2_ (50 ml/min) at 400°C for 1 h in a U-shape reactor The following catalysts were prepared: 1%Pt, 1%Pt-0.25%In, 1%Pt-0.5%In, 1%Pt-1%In, 1%Pt-1.5%In. The percentages indicate the nominal loading of Pt and In on the carbon supports. Before some characterization techniques and before the reaction tests the samples were also reduced under hydrogen flow (100 ml/min) for 1 h in a U-shape reactor.

### Catalysts Characterization

The textural properties of the prepared catalysts were determined by N_2_ adsorption at −196°C. Before the measurements, the samples were dried at 110°C for 12 h and out-gassed at 100°C under vacuum for 8 h. Their surface areas were calculated using the BET method in the relative pressure range from 0.05 to 0.25.

Experiments of temperature-programmed reduction (TPR) with H_2_ were carried out on the fresh catalysts in a U-shaped quartz cell using a 5%H_2_/He gas flow of 50 cm^3^/min, with a heating rate of 10°C/min. Hydrogen consumption was followed by on-line mass spectrometry.

X-Ray photoelectron spectroscopy was performed with a K-ALPHA spectrometer (Thermo Scientific). All spectra were collected using Al-Kα radiation (1,486.6 eV), monochromatized by a twin crystal monochromator, yielding a focused X-ray spot with a diameter of 400 μm, at 3 mA × 12 kV. The alpha hemispherical analyzer was operated at the constant energy mode with survey scan pass energies of 200 eV to measure the whole energy band and 50 eV in a narrow scan to selectively measure the particular elements. Charge compensation was achieved with the system flood gun that provides low energy electrons and low energy argon ions from a single source. The C 1s core level was used as reference binding energy, and it is located at 284.6 eV. The powder samples were pressed and mounted on the sample holder and placed in the vacuum chamber. Before recording the spectrum, the samples were maintained in the analysis chamber until a residual pressure of ca. 5 × 10^–7^ Nm^−2^ was reached. The quantitative analysis was estimated by calculating the integral of each peak, after subtracting the S-shaped background, and by fitting the experimental curve to a combination of Lorentzian (30%) and Gaussian (70%) lines. The treatment of the sample prior analysis was done *ex-situ* and conserved in octane. Suspensions were evaporated in the XPS chamber under vacuum conditions.

TEM studies were carried out using a JEOL-JEM2010 (JEOL LTD, Tokyo Japan) equipment operating at 120 kV. Sample material was mounted on a holey carbon film supported on a Cu grid by drying a droplet of a suspension of ground sample in ethanol on the grid.

CO chemisorption experiments were carried out to measure the metal dispersion of Pt. In brief, 100 mg catalyst was filled in a U-shaped quartz reactor. Before the CO chemisorption process, the catalysts were reduced by pure hydrogen at 400°C for 1 h and cooled down to 40°C in a flow of helium. Finally, the samples were saturated with pure CO at different pressure, using a volumetric set-up.

### Catalysts Evaluation

The reduction of nitrites was carried out in a semi-batch reactor, equipped with a magnetic stirrer and a thermostatic jacket, at room temperature and atmospheric pressure, and using hydrogen as reducing agent. Initially, 790 ml of deionised water and 400 mg of catalyst were fed into the reactor, the magnetic stirrer was adjusted to 700 rpm and the gas mixture of carbon dioxide and hydrogen (H_2_ + CO_2_ (1:1), flow rate = 200 cm^3^/min) was passed through the reactor during 15 min to remove oxygen; CO_2_ acts as pH buffer (pH = 5.5). After that period, 10 ml of a nitrite solution, prepared from NaNO_2_, were added in order to obtain an initial NO_2_^−^ concentration equal to 100 ppm.

Small samples were taken from the reactor for the determination of nitrite and ammonium concentrations after a defined period (300 min). Nitrite ions were determined by HPLC using a Hitachi Elite Lachrom apparatus equipped with a diode array detector. The stationary phase was a Hamilton PRP-X100 column (150 mm × 4.1 mm) working at room temperature, under isocratic conditions. The mobile phase was a solution of 0.1M NaCl:CH_3_OH (45:55). Ammonium ions were determined by potentiometry using a convenient selective electrode. pH values were also measured.

Catalyst performance was evaluated by calculating the nitrite conversion $\left(X_{NO_{2}^{-}}\right)$ and the selectivity to ammonium [$\left(S_{NH_{4}^{+}}\right)$] as:

$$X_{NO_{2}^{-}}=\frac{n_{NO_{2}^{-}i}-n_{NO_{2}^{-}}}{n_{NO_{2}^{-}i}}$$ (1)

$$S_{NH_{4}^{+}}=\frac{n_{NH_{4}^{+}}}{n_{NO_{2}^{-}}-n_{NO_{2}^{-}}}$$ (2)

where $n_{NO_{2}^{-}i}$ is the initial amount of nitrite (mmol) and are the amounts of the respective species (mmol) at time t (min). The selectivity to nitrogen (S_N2_) was calculated by difference.

## Results and Discussion

### Catalysts Characterization

#### N_2_ Adsorption Measurements

**FIGURE 1:**
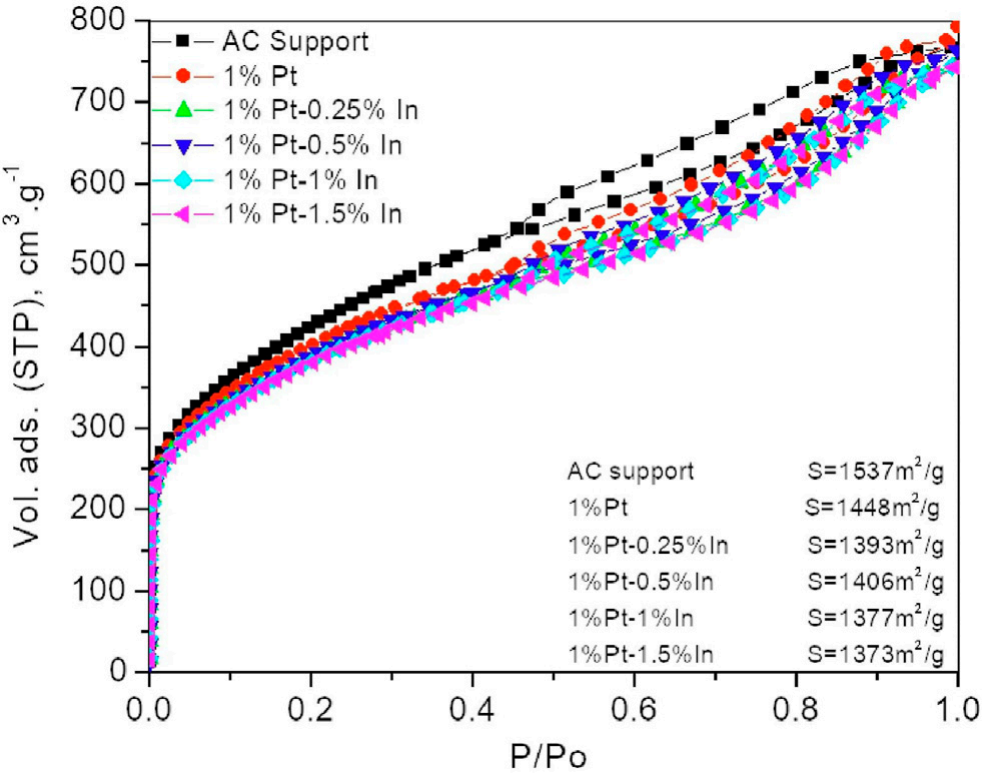
N_2_ adsorption isotherms of the different samples.

For this study, we have used a commercial activated carbon, RGC30, as catalyst support. This carbon is specially designed to be applied as catalysts support given its very low ash content, what indicates that the material is free of inorganic impurities (Gonçalves et al., 2012) that might affect the catalytic activity. Furthermore, the RGC30 carbon presents a hierarchically ordered porous structure, that would allow the reactant to diffuse in and out easily (Pérez-Ramírez, 2012). The N_2_ adsorption isotherms of the parent carbon, as well as those of the prepared catalysts after being heat-treated under flowing N_2_ at 400°C for 1 h, are shown in Figure 1. The isotherms show a steep uptake at low relative pressure indicating a large amount of micropores, and a pronounced slope in the relative pressure range from 0.2 to 0.95, which indicates the presence of a large amount of mesopores in the porous structure. If we compare the parent activated carbon with the impregnated samples, it can be seen that the amount of micropores slightly decreases after impregnation. This indicates that the metal particles are blocking a small amount of micropores. However, the surface area only decreases from 1,537 m^2^/g for the parent carbon to 1,373 m^2^/g in sample 1%Pt-1.5%In (look up at the insert). Thus, it can be concluded that the porosity of the carbon support is not dramatically modified after impregnation.

#### Temperature-programmed Reduction

**FIGURE 2:**
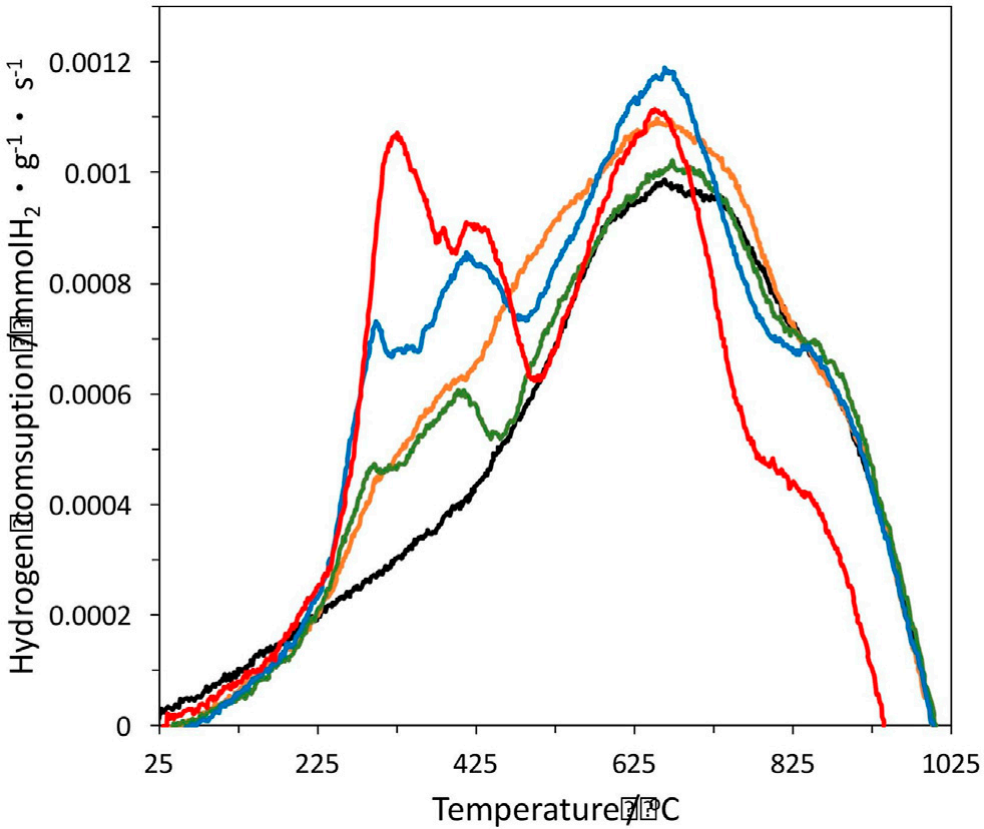
Temperature programmed reduction of the different samples. 1%Pt is in black, 1%Pt-0.25%In in orange, 1%Pt-0.5%In in green, 1%Pt-1%In in blue and 1%Pt-1.5%In in red.

The temperature-programmed reduction profiles for the samples heat-treated in N_2_ flow at 400°C for 1 h are shown in Figure 2. The monometallic 1%Pt catalyst shows a broad peak of hydrogen consumption centred at 680°C, which is associated to H_2_ chemisorption, dissociation at the platinum particles surface and subsequent spill-over onto the carbon surface. The hydrogen atoms locate at the unsaturated carbon sites generated by the thermal decomposition of the surface oxygen groups of the support. This interpretation has been confirmed by the authors’ groups (Huidobro et al., 2001; Fraga et al., 2002). The hydrogen consumption peak corresponding to the reduction of oxidized platinum species in the precursor was not detected, given that they become reduced upon the heat treatment at 400°C, as we could check by XPS analysis (see below). The heat treatment under inert conditions produces a reductive environment due to the presence of carbon.

The bimetallic catalysts show different TPR profiles. The broad band centred at 680°C is kept, and two new peaks appear at lower temperatures which are associated to the reduction of In_2_O_3_ formed upon decomposition of the indium nitrate precursor. The peak centred at 420°C is due to the reduction of In_2_O_3_ particles supported on the carbon, while the peak centred in between 290 and 320°C is ascribed to the reduction In_2_O_3_ in close proximity to Pt particles, which is favoured by hydrogen spill-over from the platinum surface (Marchesini et al., 2010). The In_2_O_3_ loading strongly affects the peak intensity, as it could be expected. Thus, the higher the In_2_O_3_ loading the higher the intensity of both peaks, this confirming that the hydrogen consumption is due to the reduction of In_2_O_3_. It is clearly shown that Pt and In_2_O_3_ particles are close together, as desired to induce the promoter effect. However, this effect depends of the oxidation state of indium, as in the metallic state it could form alloys with platinum. For the evaluation of the chemical surface composition of the catalysts, XPS analysis was carried out.

#### X-Ray Photoelectron Spectroscopy

**FIGURE 3:**
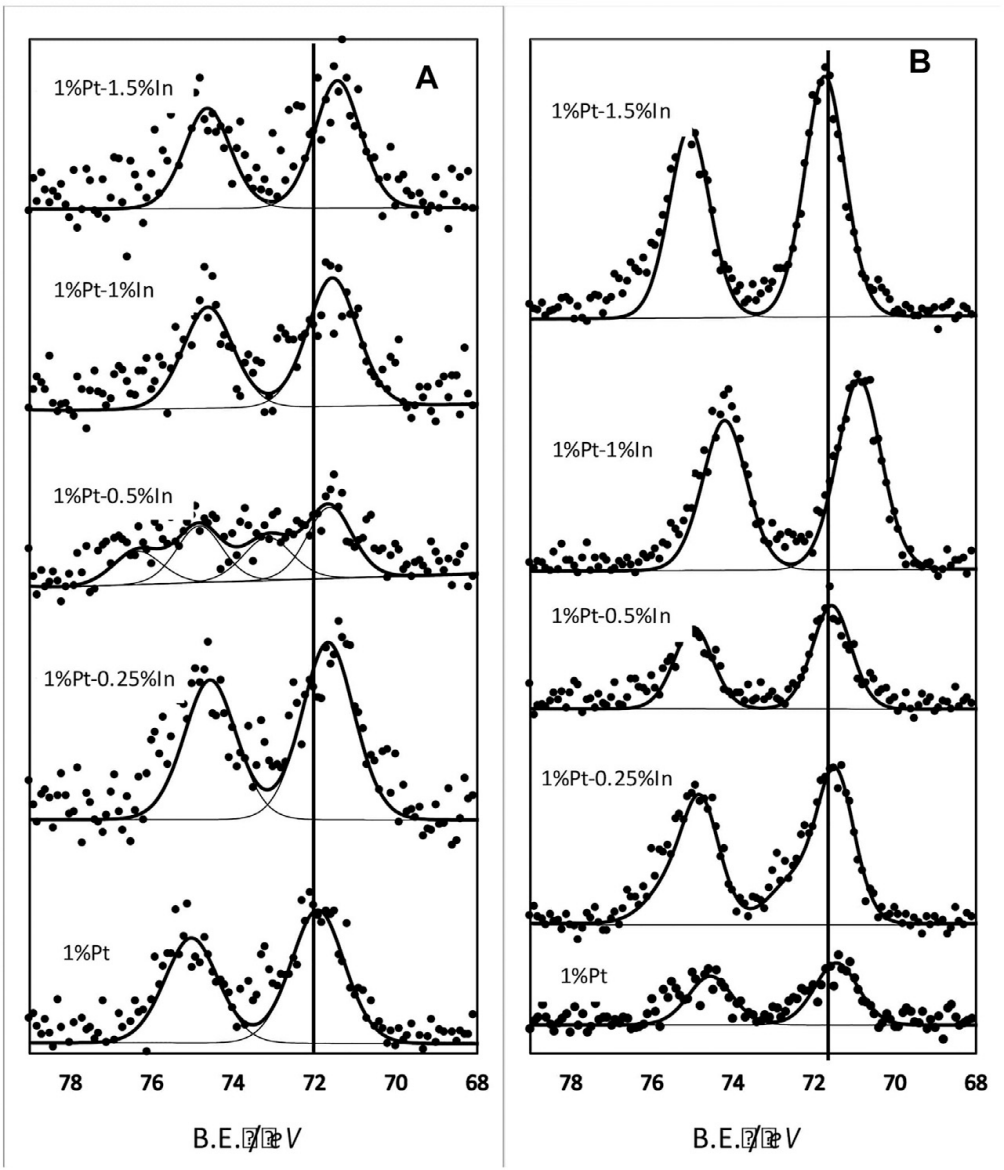
Pt 4*f* core level spectra of the different catalysts: **(A)** samples treated at 400°C in N_2_ **(B)** samples treated at 400°C in N_2_ and further reduced in hydrogen at 400°C.

Once the reducibility of the catalysts was assessed by TPR experiments, the electronic properties of the active phase and the promoter were studied by XPS measurements. Figure 3A shows the Pt 4*f* core level spectra of the different catalysts after being heat treated at 400°C under inert atmosphere. The non-promoted monometallic sample (1%Pt) shows two peaks centred at 71.9 eV (4*f*_7/2_) and 75.2 eV (4*f*_5/2_) respectively, which correspond to the spin orbit components due to the *j-j* coupling of the 4*f* region (Ramos-Fernández et al., 2009). Thus, there is only one type of platinum species in the catalyst. The 4*f*_7/2_ band is located at slightly higher binding energy that the one that can be ascribed to reduced platinum (71.5 eV) (Ramos-Fernández et al., 2008a). However, it is also located at lower binding energy than that corresponding to Pt^2+^ species (72.6 eV) (Ramos-Fernández et al., 2008b; Kundu et al., 2012). In the literature, this binding energy value is usually ascribed to small metallic platinum nanoparticles, where there is a lack of electron relaxation after the photoemission event due to the small particle size (Huidobro et al., 2002; Bera et al., 2003; Pastor-Pérez et al., 2014; Bergman et al., 2018)^.^ If fact, we also showed this phaenomenon in a previous work (Soares et al., 2012). When we look up the bimetallic samples, the Pt 4*f* peaks are shifted to lower binding energies. It clearly points that indium has an enormous effect on Pt electronic properties and confirms that both species are in close proximity, as discussed above. The shift to lower binding energies may be due to the presence of larger Pt particles that will allow an easier release of the electrons, but also to the interaction between Pt and In. If we look at the TEM images (non shown), we do see that there is not agglomeration of Pt particles and thus, the change in binding energy in the bimetallic catalysts with respect to the monometallic one must be ascribed to the modification of the platinum electronic properties by the presence of indium species. In this way, the interaction of platinum atoms with the indium species would produce an electron enrichment of the formers, which would cause the shift of the binding energy towards lower values, as observed. In fact, the Pauling electronegativity of Pt (1.78) is lower than that of In (2.28) (Wang et al., 2018). Furthermore, there is also a clear trend, as the higher the amount of In in the catalyst the larger the shifting to lower bindings energies. This proposed electron transfer will have also an effect on the In XPS spectra, as it will be discussed below.

All the promoted samples present a single type of platinum species (Pt^0^) except the 1% Pt-0.5% In catalyst, whose spectra can be deconvoluted in several peaks pointing to the existence of different Pt species. This is due to the low concentration of Pt at the surface of this particular catalyst that makes the deconvolution tricky and not straightforward. We can conclude from the analysis of the Pt 4*f* core level that Pt is electronically modified due to the strong interaction with indium.

XPS analysis was also carried out on the samples reduced in pure hydrogen at 400°C, and the results are reported in Figure 3B. Two bands appear (the Pt 4*f* doublet) located at binding energies that can be ascribed to Pt^0^. All the spectra are shifted to lower binding energies than the unreduced samples (Figure 3A), which indicates that the Pt-In interaction is enhanced when the samples are reduced. This effect is especially pronounced in catalyst 1%Pt-1%In, which is actually the most selective sample towards N_2_ in the reduction of nitrite (see results below).

**FIGURE 4:**
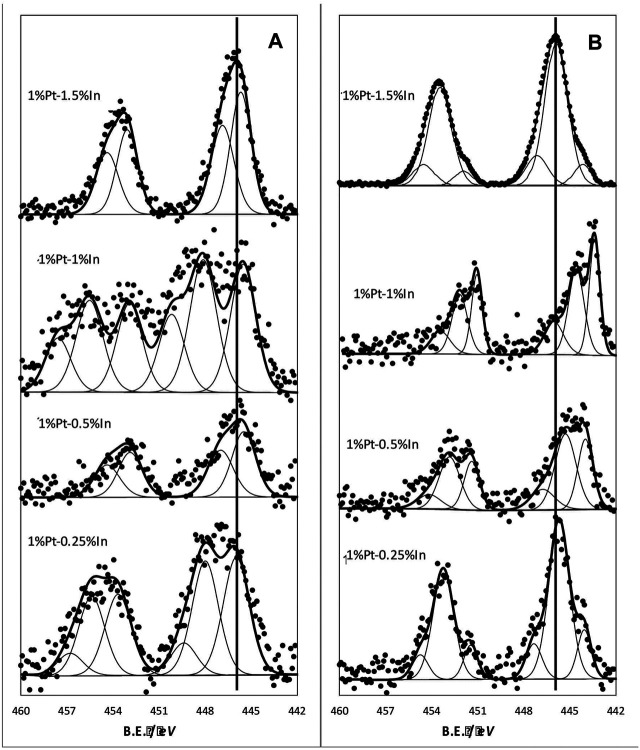
In 3*d* core level spectra of the different catalysts: **(A)** samples treated at 400°C in N_2_ **(B)** samples treated at 400°C in N_2_ and further reduced in hydrogen at 400°C.

The indium XPS 3*d* level spectra for samples treated in N_2_ at 400°C is shown in Figure 4A. The spectra are characterized by a doublet caused by the spin-orbit coupling of In 3*d*_5/2_ and 3*d*_3/2_ levels. As we can see, the peaks can be deconvoluted into several contributions, this indicating the presence of different In species. The deconvolution of the In 3*d*_5/2_ band yields two bands for catalysts 1%Pt1.5%In and 1%Pt-0.5%In, and three bands for 1%Pt-0.25%In and 1%Pt-1%In. In all cases, the lower binding energy band is above 444 eV, which is usually assigned to In^3+^ in In_2_O_3_ (Cao et al., 2014; Wang et al., 2018). This could be a consequence of the small particle size of indium oxide crystallites dispersed on the carbon support, but it could also be a proof of their interaction with the platinum particles. A more or less heterogeneous distribution of In_2_O_3_ nanocrystals, in terms of their interaction with the metal particles, yields a broad In 3*d*_5/2_ band that can be deconvoluted into several contributions.

The reduction treatment at 400°C produces important changes in the XPS In 3*d* spectra (Figure 4B). Thus, the bands are now shifted towards lower binding energies for all the samples, which indicates that In_2_O_3_ has been partially reduced upon the hydrogen treatment. We can also see that the shift is more pronounced for the sample 1%Pt-1%In, this indicating that the reducibility of In is enhanced in this sample.

It is important to take into account that the presence of metallic indium after the reduction treatment allows the formation of Pt-In alloy phases, with the consequent modification of the Pt catalytic properties. Mériaudeau et al. (1995) showed that alloy Pt_x_In_y_ phases can be prepared when working with atomic Pt:In ratios from 0.33 to 2.3, which is exactly the range used in this work. So, the possibility of having Pt-In alloys is real. Furthermore, Wegener et al. (2018) demonstrated that several type of Pt-In alloys at the nanometer scale can be formed, and they also demonstrated by using EXAFS spectroscopy that the electronic properties of the alloys strongly depended on the composition. They have also found that, as expected, the coordination number of platinum decreased with In addition. They have found that the Pt L3 white line shifted to higher energies. Filez et al. (2015) found the same behavior for Pt-In catalysts when these materials were submitted to a reduction treatment. They explained this finding in terms of electron donation from In to Pt due to their differences in electronegativity when the alloy is formed. This is pretty much in line with what we found using XPS. So, we can conclude that the alloy formation takes place in our catalysts after the reduction treatment.

Furthermore, Pašti et al. (2013) analyzed different types of Pt-In alloys using DFT, and used these alloys in the Oxygen Evolution Reaction. They showed that the way that hydrogen adsorbs in these alloys strongly depends on the composition and the final structure of the alloy. They found that the strength of the hydrogen adsorption changed with the In content due to the isolation or dissolution of Pt atoms in the alloy, which produces low coordinated Pt atoms with an increased electron density that module the adsorption of hydrogen.

Combining TPR and XPS results we can state that there is a strong interaction between platinum and indium species both in the calcined and the reduced samples. The interaction is stronger in the reduced samples (the shift to lower binding energies (XPS) is more pronounced), which is due to the formation of Pt-In alloy phases. Consequently, this strong interaction has a remarkable effect on the catalytic activity of these catalysts.

CO chemisorption has been used to assess the amount of accessible Pt sites, since it is widely reported that CO does not chemisorb on indium sites. The results obtained with samples calcined and reduced at 400°C before CO chemisorption are shown in Table 1. The monometallic sample adsorbs almost twice CO than its counterparts. Indium addition considerably decreases the number of accessible Pt surface sites, although there is not a direct correlation between the amount of indium and the decrease in CO adsorption. This observation indicates that platinum atoms are in closed proximity with In atoms, as it has been described before, and the ability to chemisorb CO decreased.

**TABLE 1:** CO chemisorption results. Table shows the Pt dispersion calculated using a CO:Pt stoichiometry 2:1.

Catalysts	Pt dispersion (%)
1%Pt	5.1
1%Pt-0.25%In	2.6
1%Pt-0.5%In	2.5
1%Pt-1%In	2.7
1%Pt-1.5%In	2.6

According to the characterization techniques used we can conclude that Pt-In alloy is formed in the reduced samples.

### Catalytic Tests

It is well established that nitrates are only reduced over bimetallic catalysts, preferably Pd-Cu catalysts, whereas nitrite can be reduced over monometallic catalysts (Soares et al., 2008; Soares et al., 2011). In the present work, the effect of the presence of indium in carbon-supported platinum-based catalysts on the activity and selectivity during the reduction of nitrites has been studied. The influence of the calcination and reduction temperatures used in the preparation of the catalysts was additionally investigated. An uncalcined catalyst (N_Cal_) and an unreduced catalyst (N_Red_) were also tested, as it had been observed in a previous work that non heat-treated Pd-Cu and Pt-Cu catalysts supported on activated carbon exhibited good catalytic performance in nitrate reduction (Soares et al., 2010).

#### Influence of Pt-In Atomic Ratio

The composition of bimetallic catalysts plays an important role in the nitrate reduction (Soares et al., 2009) in terms of both activity and selectivity, whereas in nitrite reduction it affects manly the process selectivity (Yoshinaga et al., 2002; Zhang et al., 2008). Therefore, we decided to study the influence of the Pt/In atomic ratio in nitrite reduction over samples calcined and reduced at 400°C.

**FIGURE 5:**
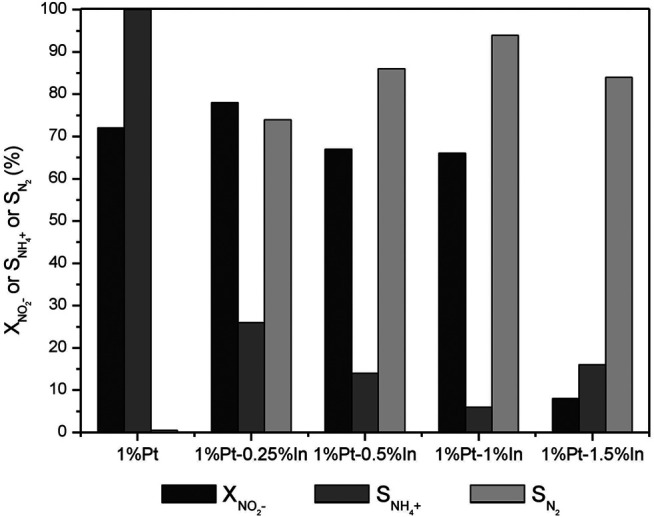
Dark grey NO_2_^−^ conversion, grey NH_4_^+^ and light grey N_2_ selectivities after 300 min of reaction on the activated carbon-supported 1%Pt-x%In/AC catalysts calcined and reduced at 400°C.

Figure 5 shows that the monometallic 1%Pt catalyst is very active for nitrite reduction. The addition of a small amount of In (0.25 wt%) slightly increases the nitrite conversion after 5 h of reaction from 72 to 78%, but a further increase of the In loading to 0.5 wt% and to 1.0 wt% slightly decreases the nitrite conversion to 67 and 66%, respectively. The catalysts with the highest amount of In (1.5 wt%) show the lowest nitrite conversion. It is generally accepted that nitrite reduction occurs on noble metal sites (Prüsse and Vorlop, 2001; Zhang et al., 2008); nevertheless, some authors (Pintar et al., 1998; Yoshinaga et al., 2002) suggest that nitrite may be converted on the surface of monometallic Pd or bimetallic Pd-Cu bimetallic sites. The results obtained in this work show that nitrite reduction occurs on the Pt sites, once the addition of indium decreases the conversion, this effect being more pronounced for the catalyst with the highest In amount. The decrease of nitrite conversion in the bimetallic catalysts with regard to their monometallic counterpart is related to the decrease of surface platinum sites due to the coverage and/or dilution of surface platinum atoms with indium species. However, the change in performance cannot be only ascribed to the Pt accessible sites, as it is also related to the electronic properties of the Pt active phase and the In promoter. XPS spectra show that the electronic properties of both In and Pt vary considerably with the catalyst composition.

The results obtained by Witońska et al. (2008) using 5%Pd-In catalysts supported on alumina are similar to those obtained in this work. They also observed that the catalyst with the highest indium content presented the lowest nitrite conversion. According to them, the different performance of the Pd-In/Al_2_O_3_ bimetallic catalyst with higher amount of indium (5 wt%) in comparison with catalysts with lower amounts (0.5–2 wt%) is related to different surface structures in those catalysts. Marchesini et al. (2008) reported that the catalytic activity decreased when the In loading was increased, which is related to a decrease of isolated Pd sites that are responsible for the dissociation of hydrogen. They claimed that the rate of each step in the reaction network is strongly dependent on the availability of hydrogen at the catalyst surface. Pašti et al. (2013) used DFT modelling to calculate that the adsorption of hydrogen is stronger in Pt alloyed with In than in monometallic Pt. However, the catalytic activity is this system is not only defined by the strength of hydrogen adsorption, but also by the amount of Pt active sites. CO chemisorption on our catalysts shows that the amount of accessible Pt strongly decreases when In is added. So, we have two factors that balance each other: the strength of hydrogen adsorption and the amount of active sites. The monometallic catalyst has more accessible active sites than the bimetallic ones and, for that reason, it is more active. The fact that the sample having the largest amount of In has very little catalytic activity must be related to the fact that In is diluting the Pt species as the surface.

Besides the decrease in the nitrite conversion, the addition of In significantly increases the selectivity to nitrogen. As it can be seen in Figure 5, the monometallic Pt catalyst converts all the nitrite into ammonium, but the addition of In significantly decreases its production. The highest nitrogen selectivity (94%) is obtained for the 1%Pt-1%In catalyst, the ammonium concentration after 5 h of reaction being only 1.5 mg/L. This result must be related to the disposition of the metals on the catalyst surface and with the electronic properties of the Pt active phase and the promoter. In the monometallic Pt catalyst, the noble metal particles are isolated and, as platinum is very active for hydrogenation reactions, the nitrite molecules are deeply hydrogenated into ammonium. In the Pt-In bimetallic catalysts, platinum and indium particles are mainly close together, with the possibility of forming alloys, which modifies the electronic properties of platinum as observed in the XPS analysis. This reduces the ability of platinum for hydrogenation, favoring the combination of nitrogen-containing intermediates in order to promote the formation of nitrogen molecules. In addition, according to the qualitative classification of Bond (Bond, 1987) for H_2_ chemisorption on metals, indium does not chemisorb appreciably hydrogen. Thus, it was expected that the hydrogen available on the catalyst surface decreases with the indium addition due to the decrease of the accessible surface area of free platinum. Miyazaki et al. (2015) reported a nitrite conversion of 31.5% over a 1 wt% Pt/Al_2_O_3_ catalyst after 190 min of reaction and only 11.1% of nitrogen selectivity. Selectivities to ammonium higher than 70% were obtained by Yoshinaga et al. (2002) and Chinthaginjala and Lefferts (2010) when monometallic palladium catalyst supported on carbon materials were used in nitrite reduction. Nevertheless, in a previous work (Soares et al., 2008) using activated carbon as support and similar experimental conditions to those used in this work, it was observed that palladium and platinum yielded nitrite conversions of 100 and 64% and nitrogen selectivities of 95 and 40%, respectively. The catalysts tested in this work offer very high nitrogen selectivities (96%), compared with the values reported in the literature, this indicating that the addition of indium increases significantly the selectivity of the process to nitrogen.

**SCHEME 1:**
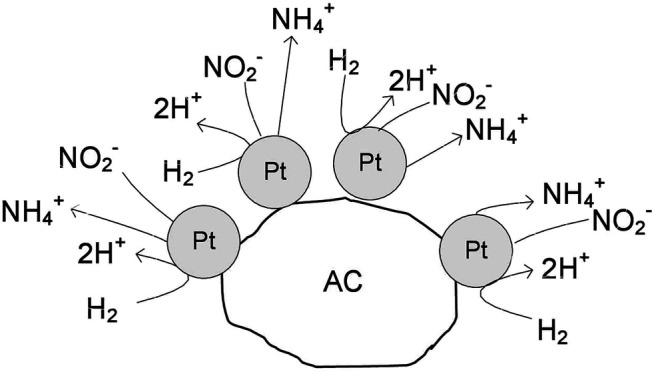
Nitrite reduction over the monometallic Pt catalyst.

**SCHEME 2:**
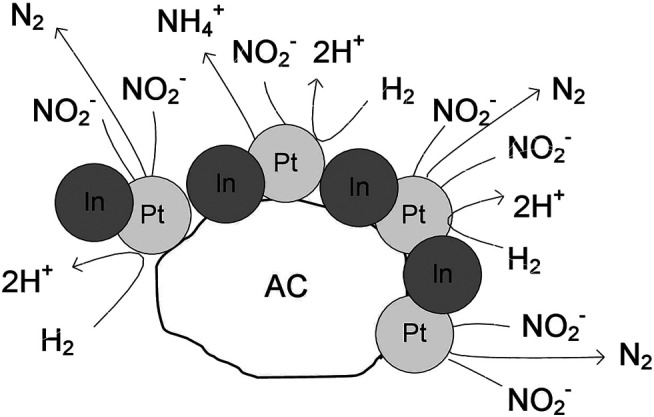
Nitrite reduction over the bimetallic Pt-In catalysts.

It can be concluded that the amount of hydrogen available on the catalyst surface controls the selectivity of the process, and it is related to the accessible surface of free platinum and the electronic properties of Pt. In the monometallic Pt monometallic (Scheme 1) the surface presents several platinum particles, which have high capacity to chemisorb hydrogen. Consequently, the formation of ammonium is favored due to the deeply hydrogenation of nitrite. In the Pt-In bimetallic catalysts (Scheme 2) the catalyst surface presents several metal particles corresponding to Pt-In alloy. With the increase of the indium content in the bimetallic catalysts, the capacity for hydrogenation is modulated by electronic properties of Pt promoted with In. Thus, in this case, the combination of the nitrogen-containing intermediates in nitrogen molecules is favored, increasing the selectivity to nitrogen. Nevertheless, independently of the type of catalyst (mono or bimetallic) the nitrite molecules are reduced on the Pt sites, what is confirmed by the decrease of nitrite conversion with the increase of the indium content.

### Influence of Pre-treatment Conditions

**FIGURE 6:**
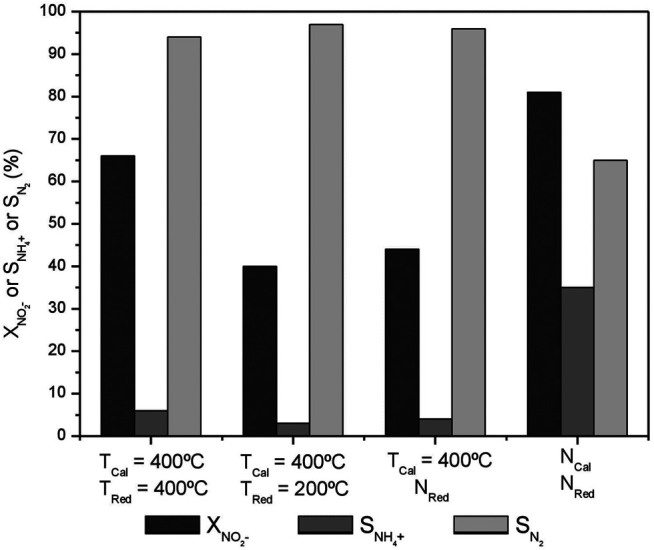
NO_2_^−^ conversion, NH_4_^+^ and N_2_ selectivities after 300 min of reaction on the activated carbon-supported 1%Pt-1%In catalyst calcined and reduced at different temperatures.

Previous studies demonstrated that the activity and selectivity of nitrate reduction are favored when low calcination and reduction temperatures (Soares et al., 2010) are used in the preparation of catalysts supported on carbon materials. Therefore, with the aim of optimizing the preparation of the carbon-supported Pt-In catalysts, the 1%Pt-1%In sample was selected (as it presents the best performance) to assess the effect of the activation variables on the reduction activity and selectivity to nitrogen. The results are reported in Figure 6.

**FIGURE 7:**
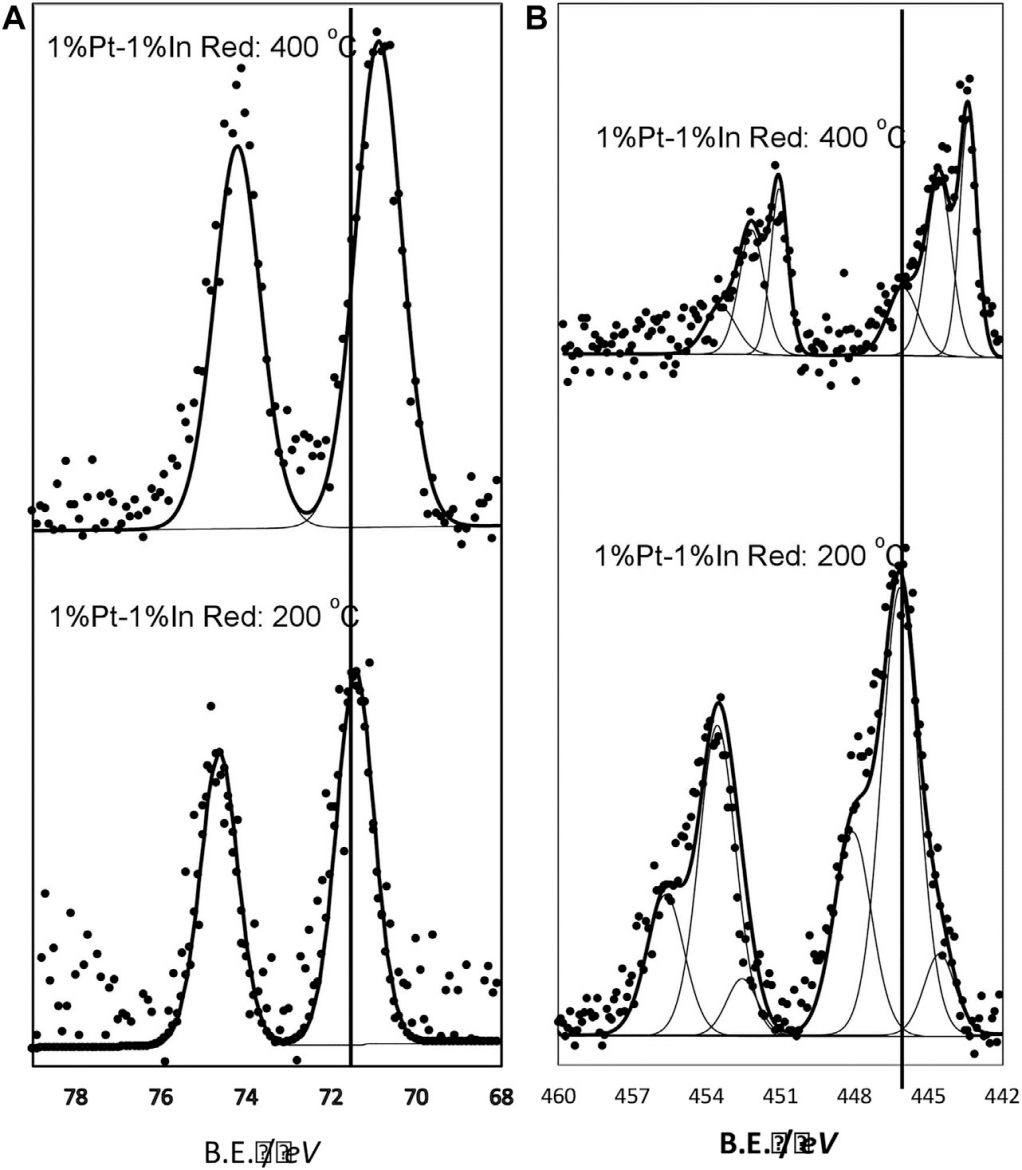
In 3*d* core level spectra **(A)** Pt 4*f* core level spectra **(B)**. Samples treated at 400°C in N_2_ and further reduced in hydrogen at 200 and 400°C.

It can be observed that the conversion attained is quite different depending on the calcination and reduction conditions. The highest nitrite conversion is obtained for the uncalcined, unreduced catalyst (N_Cal_ N_Red_), but it also presents the lowest nitrogen selectivity (65%), which must be related to the fact that indium and platinum are not interacting properly and the electronic properties of Pt and In are not modified to a large extent. The catalysts prepared with moderate temperature treatments (T_Cal_ = 400°C T_Red_ = 200°C and T_Cal_ = 400°C N_Red_) present very similar performances (40 and 44%, respectively, for nitrite conversion, and 97 and 96% for the nitrogen selectivity), with an ammonium formation below the maximum allowed concentration in drinking water. It can be concluded that the Pt-In interaction is already induced upon the calcination treatment, causing the nitrogen selectivity to increase; furthermore, the reduction treatment at 200°C does not change much this interaction. The situation is different when the catalyst is activated under harsher conditions (T_Cal_ = 400°C, T_Red_ = 400°C), when higher nitrite conversion (66%) and high nitrogen selectivity (94%) are achieved. The Pt 4*f* and In 3*d* XPS core spectra of this sample are reported in Figure 7, and compared with those of the catalyst reduced at 200°C. As it can be seen, the electronic properties of Pt and In are strongly affected by the reduction treatment and, consequently the nitrogen selectivity. The results obtained clearly show that the calcination and reduction temperatures strongly affect the catalyst performance, being this effect more pronounced in the nitrite conversion.

It should be noticed that the initial nitrite concentration (100 mg/L) used in this study is very high compared with the nitrite concentrations normally present in groundwater, which are relatively low (normally < 1 mg/L)^1^. Nevertheless, the results obtained allow to conclude that the quality standards for drinking water can be obtained for these low concentrations normally found in groundwater due to the low amount of ammonium formed after reduction of nitrite in the presence of most of the catalysts considered in the present work. In our optimization experiments it was observed for all the catalysts tested that, after a stabilization period of about 30 min of reaction, the selectivities remained practically constant and did not change with conversion. So, it is expected that the nitrogen selectivities remain high, independently of the initial nitrite concentration.

## Conclusion

Pt-In bimetallic catalysts were studied in the catalytic reduction of nitrite. The increase of the indium content up to 1 wt% significantly increases the selectivity to nitrogen due to the decrease of the accessible surface of the noble metal atoms and the modification of the electronic properties of both the noble metal and the promoter, this change in electronic properties must be ascribed to the formation of Pt-In alloy. For higher In loadings the nitrite conversion significantly decreases. In the bimetallic catalysts, indium and platinum metal particles are interacting and forming alloy phases that favour the combination of nitrogen-containing intermediates to produce N_2_ instead of being deeply hydrogenated into ammonium. The nitrogen selectivity increases with the decrease of the available hydrogen on the catalyst surface, which is achieved using high In loadings. The calcination and reduction temperatures play an important role in the activity, although they do not have an important effect on the nitrogen selectivity.

## Data Availability

The original contributions presented in the study are included in the article/Supplementary Material, further inquiries can be directed to the corresponding author.
